# Proficiency of phenotypic drug susceptibility testing for *Mycobacterium tuberculosis* in China, 2008–2021

**DOI:** 10.1371/journal.pone.0304265

**Published:** 2024-05-29

**Authors:** Yuanyuan Song, Bing Zhao, Shengfen Wang, Yang Zheng, Yang Zhou, Xichao Ou, Hui Xia, Yanlin Zhao

**Affiliations:** National Center for Tuberculosis Control and Prevention, Chinese Center for Disease Control and Prevention, Beijing, the People’s Republic of China; Rutgers Biomedical and Health Sciences, UNITED STATES

## Abstract

To analyze the results of proficiency testing for anti-tuberculosis drug susceptibility testing (DST) in China. Number of laboratory participating the proficiency testing performed DST, and the sensitivity, specificity, reproducibility, and accordance rate were calculated from data of 13 rounds proficiency testing results for DST from 2008 to 2021. A total of 30 and 20 strains of *Mycobacterium tuberculosis* with known susceptibility results were sent to each laboratory in 2008 to 2019, 2020 and 2021, respectively. The number of participating laboratories ranged from 30 in 2009 to 546 in 2021. L-J DST was the predominant method. The specificity presented relatively higher than sensitivity. Improvement of specificity were observed for all drugs through the years, while sensitivity did not show improvement for amikacin and capreomycin. Accordance rate of pyrazinamide and kanamycin and reproducibility of capreomycin and pyrazinamide were not significantly improved through the years. Most of the participating laboratories significantly improved the quality of their DST through the consecutive rounds of proficiency testing except for second-line injectable drugs and pyrazinamide. The results highlight the importance of developing novel and/or improving existing methods for phenotypic DST for certain drugs.

## Introduction

Drug-resistant Tuberculosis (TB) remains a major public health concern in China. There were an estimated 33 000 (27,000–39 000) Multi-resistant/Rifampin-resistant Tuberculosis (MDR/RR -TB) cases in 2021 in China. Among which, 16,766 cases were detected, accounting for 50.8% of the estimated MDR/RR-TB cases [1]. Over the past years, most of provincial and city level TB laboratories established the capacity of drug susceptibility testing (DST) either by molecular or phenotypic tools. Therefore, further expansion of access to these tools and improvement of quality are more essential. Rapid molecular tests have been recommended as initial diagnostic tool. Phenotypic DST is still useful in cases who are highly suspected resistant but tested susceptible by molecular method, and to detect susceptibility to second-line drugs which are not covered by initial molecular tools. Establishment of TB laboratory network to assess phenotypic DST’s proficiency is critical. Proficiency testing uses an interlaboratory comparison to assess the performance of a laboratory tests. The nationwide DST proficiency testing (DST-PT) organized by National Tuberculosis Reference Laboratory started since 2008. Most of previous studies on proficiency testing focused on first-line drugs [2–5], but very few on second-line drugs [6,7]. There has been lack of proficiency data covering both first- and second-line drugs especially in a single country. This study retrospectively analyzed phenotypic DST proficiency testing over ten years for both first- and second-line drugs in a high TB burden country.

## Materials and methods

### Participating laboratories

Thirteen rounds of DST-PT were implemented from 2008 to 2021 except for 2015. Laboratory number participating the DST-PT increased from 30 (round 1) to over 500 laboratories (round 13), among which the city-level laboratories accounted for the most (Table 1).

**Table 1:** Laboratories participating the DST proficiency testing in each round.

Round (year)	No. laboratories (RFP,INH,EMB,SM)				No. laboratories (PZA)				No. laboratories (KM,AK,CPM,OFX)			
	Provincial level	City level	County level	Total	Provincial level	City level	County level	Total	Provincial level	City level	County level	Total
**1 (2008)**	25	8	0	33	0	0	0	0	12	0	0	12
**2** **(2009)**	18	12	0	30	0	0	0	0	11	2	0	13
**3** **(2010)**	26	32	0	58	0	0	0	0	11	16	0	27
**4** **(2011)**	30	24	0	54	0	0	0	0	28	23	0	51
**5** **(2012)**	31	110	0	141	0	0	0	0	31	105	0	136
**6** **(2013)**	35	190	0	225	0	0	0	0	35	183	0	218
**7** **(2014)**	41	295	1	337	13	10	0	23	41	287	1	329
**8** **(2016)**	41	351	1	393	13	18	0	31	41	345	1	387
**9** **(2017)**	48	369	11	428	9	19	5	33	48	367	11	426
**10** **(2018)**	58	381	18	457	9	18	7	34	54	380	18	452
**11** **(2019)**	56	411	20	487	8	26	3	37	56	407	13	476
**12** **(2020)**	56	413	29	498	7	31	4	42	54	406	20	480
**13** **(2021)**	67	448	31	546	7	26	1	34	65	439	20	524

Aberrations: RFP, rifampin; INH, isoniazid; EMB, ethambutal; SM, streptomycin; PZA, pyrazinamide; KM, kanamycin; AK, amikacin; CPM, capreomycin; OFX, ofloxacin.

### Origin and composition of proficiency testing panels

Each DST-PT panel was from the World Health Organization (WHO)’s coordinating Belgium supernational reference laboratory (SRL) or Hong Kong TB SRL. DST using Lowenstein-Jensen (L-J) medium and liquid Mycobacterium Growth Indicator Tube (MGIT) was performed in China’s National Tuberculosis Reference Laboratory (NTRL). Strains were sub-cultured and allocated into 2.0 ml plastic cryovials containing L-J medium or Middlebrook 7H9 with glycerol before transportation to 31 Provincial TB Reference laboratories (PTRLs). PTRLs sub-cultured strains further, and transported the strains to city- or county-level laboratories. The definition of provincial-, city-, and county-level laboratory was based on China’s administrative divisions. That is, the medical institution to which the laboratory affiliated is defined as the corresponding level. Approval from relevant department is required before transportation. Triple packaging compliment with UN packing instruction P620 was used to transport panel strains either from NTRL to provincial level labs or from which to lower level labs. Panel strains were transported either by air flight by company with qualifications for transporting materials containing infectious substances or by trained staffs using vehicles (S1 Fig).

A total of 30 strains consisting of 10 pairs of duplicate strains and 10 single strains were used in 1–11 rounds, while 20 strains composed of 9 duplicate strains, one single MTB, and one single Nontuberculous Mycobacteria (NTM) isolate in the last two rounds (Table 2). 1–3 NTM strains were added for strain identification since the 9^th^ round. The types of drugs referred to the proficiency testing provided by supernational laboratory network in the same period.

**Table 2:** Proficiency testing panels composition in each round.

Round	DST result	RFP	INH	EMB	SM	PZA	KM	AK	CPM	OFX
**1**	R	13	11	15	16	—	11	7	9	9
	S	17	19	15	14	—	19	23	21	21
**2**	R	12	16	9	12	—	11	7	9	9
	S	18	14	21	18	—	19	23	21	21
**3**	R	9	19	12	20	—	11	7	9	9
	S	21	11	18	10	—	19	23	21	21
**4**	R	12	20	14	14	—	11	7	9	9
	S	18	10	16	16	—	19	23	21	21
**5**	R	10	19	12	13	—	5	3	3	4
	S	20	11	18	17	—	25	27	27	26
**6**	R	13	20	16	17	—	9	8	8	8
	S	17	10	14	13	—	21	22	22	22
**7**	R	19	19	13	17	11	9	7	7	11
	S	11	11	17	13	18	21	23	23	19
**8**	R	17	18	10	17	15	10	6	8	7
	S	13	12	20	13	13	20	24	22	23
**9**	R	19	20	12	—	11	12	12	8	11
	S	10	9	17	—	18	17	17	21	18
	NTM	1	1	1	—	1	1	1	1	1
**10**	R	17	17	11	—	14	11	8	9	10
	S	12	12	18	—	15	18	21	20	19
	NTM	1	1	1	—	1	1	1	1	1
**11**	R	18	19	14	—	13	11	11	11	11
	S	9	8	13	—	14	16	16	16	16
	NTM	3	3	3	—	3	3	3	3	3
**12**	R	11	13	10	—	10	8	6	—	—
	S	8	6	9	—	9	11	13	—	—
	NTM	1	1	1	—	1	1	1	—	—
**13**	R	6	10	—	—	5	8	8	—	—
	S	13	9	—	—	14	11	11	—	—
	NTM	1	1	—	—	1	1	1	—	—

Aberrations: R, resistant; S, susceptible; NTM, non-tuberculous Mycobacteria; RFP, rifampin; INH, isoniazid; EMB, ethambutal; SM, streptomycin; PZA, pyrazinamide; KM, kanamycin; AK, amikacin; CPM, capreomycin; OFX, ofloxacin.—represent the proficiency testing did not include the drug.

### Identification and drug susceptibility testing methods

The participating laboratories are required to use their routine used DST methods for proficiency testing, such as L-J, MGIT, and commercial minimal inhibitory concentration (MIC) method. Detailed information using different methods were summarized in Table 3. Critical concentrations for L-J and MGIT were recommended according to the WHO’s guideline (Table 4). Cutoff values of MIC method were based on manufacture’s instruction. Since the 9^th^ round, identification by biochemical test or rapid immunochromatographic assay (such as Capillia TB) was required before the DST.

**Table 3:** Number of participating laboratories using different DST methods.

Round	Method*			Total
	L-J	BACTEC MGIT	MIC	
**1**	33	0	0	33
**2**	30	0	0	30
**3**	58	0	0	58
**4**	54	0	0	54
**5**	141	0	0	141
**6**	225	0	0	225
**7**	337	23	0	360
**8**	393	31	0	424
**9**	390	33	5	428
**10**	409	38	10	457
**11**	426	45	16	487
**12**	421	48	29	498
**13**	451	49	46	546

*A few laboratories that use more than one method counted multiple times accordingly.

**Table 4:** Critical concentrations recommended for proficiency testing.

Drug	L-J (ug/mL)	MGIT (ug/mL)	MIC(ug/mL)
**RFP**	40	1	1/2
**INH**	0.2	0.1	0.1/0.2/0.25/0.4
**EMB**	2	5	2.5/4/5
**SM**	4	1	1/2
**PZA**	—	100	—
**KM**	30	2.5	1.25/2/2.5/5
**AK**	30	1	1/1/25/2.5/4
**CPM**	40	2.5	2/2.5
**OFX**	2/4*	2	1.5/2

*The critical concentration of OFX was changed from 2ug/mL to 4ug/mL for L-J medium DST at 10th and 11th round.

Aberrations: RFP, rifampin; INH, isoniazid; EMB, ethambutal; SM, streptomycin; PZA, pyrazinamide; KM, kanamycin; AK, amikacin; CPM, capreomycin; OFX, ofloxacin.

Contaminated strains will be decontaminated using 4% NaOH, subcultured and subjected to the following tests. If contamination is found to be concentrated on a specific strain, provincial labs may decontaminate and resent that strain. If the contamination cannot be resolved, they will report the results as contamination, and this strain will be excluded when calculating the performance indicators by NTRL as described in the data analysis section.

### Data analysis

Results were compared with consensus results, defined as at least 80% concordant “susceptible” or “resistant” between all reported results as previously published [3]. The performance to detect true resistance (rate of detection of judicially resistant strains, RD; sensitivity), true susceptibility (rate of detection of judicially susceptible strains, SD; specificity), intralaboratory agreement between duplicate strains (reproducibility), and accordance rates (number of correct results divided by total number of results excluding contamination strains, AR; efficiency) were calculated. If result was unavailable for one of the paired strains due to contamination or no growth, the pair was excluded from the analysis for reproducibility.

SAS software version 9.4 was used for statistical analysis. The chi-square test was used to analyze the significance of difference among rounds, and the Cochran—Armitage trend test was used to describe the trend of improvement, significant difference was defined as P<0.05.

### Ethical review

The present study is based on routine work and only involved laboratory testing of mycobacteria, not related to the individual human subjects and information, thus the ethical review was waived.

## Results

All rounds of proficiency testing implemented smoothly, except for the 3^rd^ round in which contamination of some strains occurred frequently, e.g., 58.62% (34/58) of the laboratories reported 1–5 contaminated strains, No. 1 strain was contaminated in 15.38% (4/26) provincial laboratories and 43.75% (14/32) city level laboratories, No. 30 strain was contaminated in 3.85% (1/26) provincial laboratory and 37.50% (12/32) city level laboratories.

### Accordance rate

**Fig 1:**
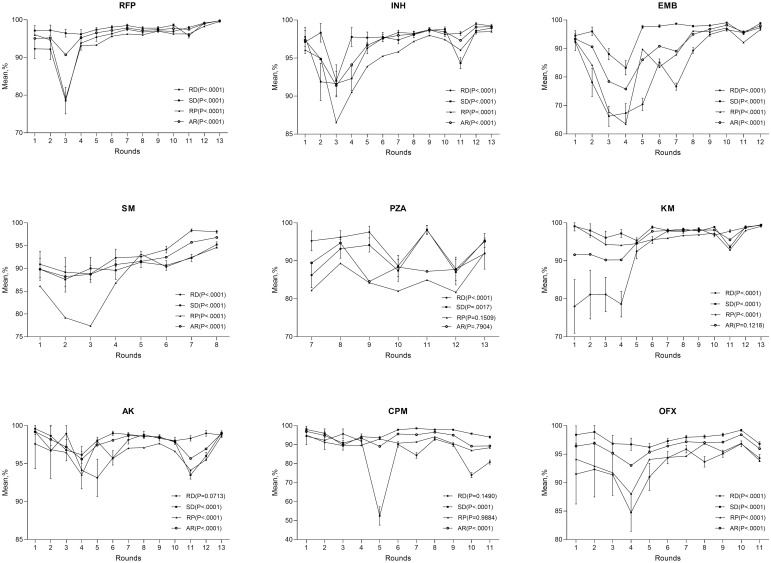
Performance of DST proficiency testing for each drug in different rounds. The mean value of RD and SD with 95% confidence intervals and mean value of RP, and AR is shown. Difference of performance among different rounds is based a Cochran-Armitage trend test. RD, rate of detection of resistant strains; SD, rate of detection of susceptible strains; RP, reproducibility; AR, rate of accordant results among total test results.

The average accordance rates of RFP, INH, EMB, SM, PZA, KM, AK, CPM and OFX were respectively 90.72 to 99.76%, 91.40 to 99.13%, 75.78 to 98.20%, 88.20 to 96.80%, 84.57 to 95.14%, 90.23 to 99.42%, 95.59 to 99.20%, 89.08 to 97.00% and 93.01 to 98.41% among all rounds (Fig 1). The accordance rate showed a significant improvement throughout all rounds (*p*<0.001) for most of drugs except for PZA (*p* = 0.7904) and KM (*p* = 0.1218) (Fig 1). Detailed values for each drug of Fig 1 were shown in S1 File. Detailed data for each participating laboratory to calculate average accordance, sensitivity, specificity, reproducibility and 95%CI was shown in S2 File.

### Sensitivity

The average sensitivity with 95% confidence intervals (CIs) of RFP, INH, EMB, SM, PZA, KM, AK, CPM and OFX ranged respectively from 78.54% (95%CI: 75.02–82.07) to 99.73% (95%CI: 99.55–99.90), 91.65% (95%CI: 90.02–93.28) to 99.50% (95%CI: 99.33–99.68), 66.24% (95%CI: 62.72–69.75) to 97.32% (95%CI: 96.87–97.77), 88.79% (95%CI: 86.98–90.61) to 98.34% (95%CI: 98.01–98.67), 87.86% (95%CI: 84.73–90.98) to 98.08% (95%CI: 96.91–99.26), 78.00% (95%CI: 70.93–85.07) to 99.48% (95%CI: 99.26–99.69), 93.14% (95%CI: 90.68–95.59) to 98.99% (95%CI: 98.62–99.35), 52.45% (95%CI: 47.61–57.30) to 95.68% (95%CI: 93.07–98.28) and 84.75% (95%CI: 81.46–88.04) to 96.81% (95%CI: 96.30–97.32) over ten years (Fig 1). For RFP, the sensitivity was higher than 90% in all rounds except for the 3^rd^ round. For INH, sensitivity was higher than 90% in 10 rounds except for round 2–4. For EMB, the sensitivity was lower than 80% in 2^nd^, 3^rd^, 4^th^, 5^th^, 7^th^ and 8^th^ round and maintain at higher than 90% since round 9. For SM, 2/8 rounds showed sensitivity lower than 90%. For KM, the sensitivity of the first four rounds was around 80%, and higher than 90% in other rounds. For AK, the sensitivity was higher than 90% in all rounds, but showed the instability between different rounds. For CPM, the sensitivity also varied a lot, especially in round 5, 10 and 11. For OFX, the sensitivity was higher than 90% except for round 4 with 84.75%. The sensitivity was significantly different between rounds for all drugs (*p*<0.001). As a whole, the sensitivity of most of drugs had a trend of improvement (*p*<0.001) from 1^st^ through 13^th^ round except for AK (*p* = 0.0713) and CPM (*p* = 0.149) (Fig 1).

### Specificity

The average specificity with 95% CIs of RFP, INH, EMB, SM, PZA, KM, AK, CPM and OFX ranged respectively from 95.79% (95%CI: 95.19–96.38) to 99.73% (95%CI: 99.61–99.85), 92.01% (95%CI: 89.90–94.11) to 98.94% (95%CI: 98.66–99.23), 83.22% (95%CI: 80.73–85.71) to 99.08% (95%CI: 98.87–99.28), 87.59% (95%CI: 84.81–90.37) to 95.24% (95%CI: 94.66–95.83), 86.23% (95%CI: 82.91–89.55) to 98.21% (95%CI: 97.11–99.31), 93.60% (95%CI: 93.05–94.15) to 99.34% (95%CI: 99.13–99.55), 93.51% (95%CI: 92.96–94.07) to 99.60% (95%CI: 98.86–100.00), 89.68% (95%CI: 87.13–92.23) to 98.57% (95%CI: 98.31–98.84) and 96.21% (95%CI: 95.58–96.84) to 99.21% (95%CI: 99.02–99.39). For EMB, the specificity showed lower than 90% in round 3 and 4. For SM, the specificity was lower than 90% in round 2. For PZA, the specificity was lower than 90% in round 7, 10, 12. The specificity was significantly different between rounds for all drugs (*p*<0.001). As a whole, the specificity for all drugs showed improvement throughout all rounds (*p*<0.05) (Fig 1).

### Reproducibility

The average reproducibility of RFP, INH, EMB, SM, PZA, KM, AK, CPM and OFX ranged respectively from 79.11% to 99.57%, 86.55% to 98.49%, 63.46% to 97.22%, 77.37% to 94.60%, 81.75% to 92.16%, 92.89% to 99.20%, 93.52% to 99.20%, 86.98% to 95.00% and 88.01% to 96.88% (Fig 1). The reproducibility of EMB was lower than 90% in round 2, 3, 4, 5, 6 and 7. The reproducibility of SM was lower than 90% in the first four rounds. For PZA, the reproducibility was lower than 90% in almost all rounds except for the 13^th^ round. For CPM, the reproducibility was lower than 90% in round 10 and 11. There were significant difference between rounds for all drugs (*p*<0.001) (Fig 1). The reproducibility also improved for most of drugs (*p*<0.001) except for CPM (*p* = 0.9884) and PZA (*p* = 0.1509) from the 1^st^ to the last round.

## Discussion

Proficiency testing is one of critical elements of quality assurance, through which the laboratory can find the major problems of DST. The present study showed that numbers of laboratory with capacity of phenotypic DST expanded rapidly in China over past years. The quality of DST for most tested drugs also improved. Provincial and city-level laboratory with phenotypic DST capacity was established as one of objectives in the 10^th^ five-year program (2011–2015) for tuberculosis control and prevention in China, which promoted the rapid increase of number of city-level laboratories providing the DST service since 2011. The performance in round 3 and 4 were worse than those of round 1 and 2, and then improved in the subsequent rounds. We think that this result is related to a higher proportion of provincial laboratories in the first two rounds, most of which participated in the drug resistance surveillance supported by WHO and global fund since 1994 [8–11], and thus had more proficiency than city level laboratories without DST experience at the earlier rounds.

For RFP and INH, the two most important first-line anti-tuberculosis drugs, all performance indicators showed good results except in round 2, 3 and 4. For EMB, the sensitivity and reproducibility did not show good results especially in earlier rounds. Since most EMB resistance related mechanisms confer only modest MIC increases and result in a significant overlap with the wild type strains MIC distribution [12], thus the current binary DST results make it difficult to distinguish this overlap. In addition, the defined critical concentration is very close to the MIC required to achieve anti-mycobacterial activity, increasing the probability of misclassification of susceptibility or resistance, and bring poor reproducibility of phenotypic DST results [13]. From 2018, WHO does not recommend EMB DST as routine testing method any more [14]. The improvement measures has been explored, such as an inconclusive MIC breakpoint of 4ug/mL was introduced for EMB in clinical and laboratory standards institute (CLSI) document, commenting that an MIC of 4ug/mL obtained by broth microdilution does not correlate with either susceptible or resistant result, and suggest repeating testing using other method (e.g., genotypic or an alternative broth method), which bring out other issues to be urgently resolved, such as the reliability of current molecular-based EMB resistance detection tools and phenotypic methods on the current critical concentration in L-J and MGIT medium. PZA was only included in the proficiency testing since the 7^th^ round in 2013. MGIT is the single phenotypic method that can be used to detect PZA resistance, limiting the rollout of PZA DST. The specificity may be affected by an over-inoculation or non-homogeneous bacterial suspension, which thus result in reducing the PZA effect due to the increased pH [15,16]. Non-homogeneous bacterial suspension, such as large clumps in the bacterial suspension when adjusting the turbidity, can also cause varied true bacterial count in the suspensions and thus affect the reproducibility. In the present study, although the sensitivity and specificity of PZA improved, the specificity remained being below than 90% without significant improvement. The specificity problems was also reported by other study on the proficiency testing against pyrazinamide [17]. The critical concentration itself may also result in inconsistent results for isolates with a PZA MIC close to this concentration [18,19]. So the phenotypic DST against PZA should be further optimized given the weakness shown in this study. DST-PT for SM was cancelled since round 9, consistent with WHO’s drug profile, based on the fact that SM was only to be considered only if AK cannot be used and the unreliability of performance even in the supranational tuberculosis reference laboratories, in which the sensitivity and specificity showed high variability [20]. For second-line injectable drugs, the best sensitivity was observed for AK, while CPM did not show improvement even after implementation of around ten years. The specificity and reproducibility of these three second-line drugs showed good performance. AK is now classified as one of Group C drugs recommended for the treatment of RR-TB, is only to be considered if DST results confirm susceptibility as in WHO guideline. OFX showed good performance, similar conclusion with other study results [20], although testing of OFX is not recommended as it is no longer used for treating resistant TB and laboratories should transition to testing the later generation FQs, such as LFX and MFX. Other studies also pointed out that except for INH and RMP, the accuracy and reproducibility of the other 7 drugs are poor [3,17,20–23].

From the implementation perspective, the contamination occurred more concentrated in laboratories of certain provinces in round 3, which indicated that the further subculture in provincial level laboratories can bring the risk of more contamination especially in the early years when the technology of provincial laboratory staff was not competent and proficient. After practice and training, the proficiency improved. DST training in China adopts a hierarchical training approach considering the large number of labs and staffs, that is, from national level to provincial level, and from provincial level to city and county level. In addition to training, it is also required that laboratories with false susceptible or false resistant results should identify the root cause, and solve the problem. Afterwards, contamination occured with very few frequency. The confidence interval of the first five rounds is relatively wide, reflecting the poor performance of the participating laboratories. On the one hand, the number of participating laboratories is small, and on the other hand, it is also related to the instability of drug results and the proficiency of staff. Although the range is wide, most of the lowest points are more than 80%, but the sensitivity of the following four drugs: RFP EMB KM CPM is less than 80%. In addition to the above reasons, it is also related to the ratio of strains (drug resistance/sensitivity). There are too few drug-resistant strains in each round, generally 3–11 strains. In particular, the number of strains resistant to CPM in the fifth round is only 3, and the total sensitivity of CPM in the fifth round is 52.45%. If there is a wrong drug resistance detection of one strain, the sensitivity is 66.67% (2/3), which reduces the detection sensitivity of CPM, and it is not because of the laboratory detection ability that this indicator is low. Similarly, the low detection rate and reproducibility rate of RFP resistance in the third round were also related to this reason (9 resistant strains). Therefore, it is also suggested that the laboratory that organizes the proficiency test of drug sensitivity test should pay attention to the stability of the strains and the number of drug-resistant strains when preparing the test strains. WHO and the International Federation against Tuberculosis and Pulmonary Disease recommend that each drug in the test strains should contain 50% of drug-resistant strains [4]. However, it is more difficult than before to constitute 50% of resistant strains for all tested drugs which include critical first-line, second-line drugs, and even new or repurposed drugs that has very low resistance rate up to now, such as bedaquiline, and Linezolid in only 10 strains. So more research on how to constitute of panels has to be explored.

It should also be noted that the proficiency test showed good results, which was related to the fact that most of the strains selected by WHO were far from the MIC [3], excluding exclude mixtures of strains and heteroresistance and the judicial result gold standard used, and so there was a certain difference from the daily clinical strains. Thus enhanced internal quality control was recommended to ensure routine DST services. MIC results as a potential phenotypic DST method are more useful than categorical DST results to classify resistance mutations. Phenotypic DST is still a useful method to detect drug resistance, which cannot be obtained by current commercial molecular based tools, such as XpertMTB/RIF, XpertMTB/RIF Ultra, Line-probe assay recommended by WHO, and some Chinese local products (Genechip MDR-TB detection assay and Melt-curve drug resistance detection assay).

In addition to the improving technical performance indicators, there are also some improvements at the implementation level over the past years of proficiency testing. The DST methods, measures taken for reduce contamination, and data analysis method have all been improved (S1 Table). However, the results report still relies on manual input through Excel spreadsheets and email, thus a more convenient information platform needs to be established.

This study also has certain limitations, the judicial results were used to evaluate the proficiency of laboratories, the MIC and genotypic results were not obtained to accurately analyze the false results which may be due to the low-level resistance. We need to determine of the minimal inhibitory concentration and characterize molecular resistance mutations to select representative and stable strains in the future [24] and this additional information can aid in resolving discrepant results and indicate future directions for proficiency testing. Second, although MIC method was used in some laboratories, there is no recommended critical concentration. The standards and quality of commercial MIC plate are various. Thirdly, the types of drugs did not contain some of critical drugs composed of MDR/RR-TB treatment regimen, such as moxifloxacin, BDQ, LZD, CFZ, DLM, which has been added in the proficiency test since 2022 and will be systematically analyzed in the future.

## Conclusions

Most of the participating laboratories significantly improved the quality of their DST through the consecutive rounds of proficiency testing. Thus, the current program of DST-PT should be continued with drugs and methods updated. All laboratories conducting DST are encouraged to participate in annual proficiency testing and to strengthen internal quality control. The reliability of some drugs still need to be resolved, however.

## Supporting information

S1 Fig Flowchart of proficiency testing of drug susceptibility testing in China. The flowchart contained laboratory testing, result reporting and feedback process. Identification test included biochemical test or rapid immunochromatographic assay (such as Capillia TB) is required to confirm MTB before DST since the 9th round. The standard results report form based on Excel is used to enter and report results from lower level labs to upper level labs as shown by green line. Excel software is used and then transferred to python since the 12th round for analysis of the performance indicators by NTRL and feedback the final performance results to provincial level labs. The provincial labs will feedback the results to lower level labs as shown by blue line. NTRL: National Tuberculosis Reference Laboratory; MTB: Mycobacterium Tuberculosis; NTM: Non-Tuberculous Mycobacteria; DST: Drug susceptibility testing.
(TIF)

S1 File Detailed values for each drug of Fig 1. (XLS)

S2 File Raw results from each laboratory for proficiency testing of 1–13 rounds of DST. (XLSX)

S1 Table List of improvement and challenges over the past proficiencity testing. (XLSX)

## References

[1] World Health Organization. Global Tuberculosis Report 2022. Geneva: WHO; 2022. https://www.who.int/publications/i/item/9789240061729

[2] Bai G-H, Kim S-J, Chang CL, Members of National and Regional Tuberculosis Reference Laboratories. Proficiency analysis of drug susceptibility testing by national-level tuberculosis reference laboratories from 1995 to 2003. J Clin Microbiol. 2007;45: 3626–3630. doi: 10.1128/JCM.00784-07 17855570 PMC2168479

[3] Van Deun A, Wright A, Zignol M, Weyer K, Rieder HL. Drug susceptibility testing proficiency in the network of supranational tuberculosis reference laboratories. Int J Tuberc Lung Dis. 2011;15: 116–124. 21276307

[4] The Committee for Mycobacterial Examinations, the Japanese Society for Tuberculosis. Evaluation standard of external quality assessment programme for drug susceptibility testing of mycobacterium tuberculosis in Japanese laboratories: proficiency testing in 2004–2010. Kekkaku. 2015;90: 481–490. 26489152

[5] Wu M-H, Chiang C-Y, Deng Y-M, Wang T-F, Jou R. Proficiency of drug susceptibility testing for Mycobacterium tuberculosis in Taiwan, 2007–2011. Int J Tuberc Lung Dis. 2013;17: 113–119. doi: 10.5588/ijtld.12.0521 23232011

[6] Jiang G-L, Chen X, Song Y, Zhao Y, Huang H, Kam KM. First proficiency testing of second-line anti-tuberculosis drug susceptibility testing in 12 provinces of China. Int J Tuberc Lung Dis. 2013;17: 1491–1494. doi: 10.5588/ijtld.13.0275 24125456

[7] Hillemann D, Hoffner S, Cirillo D, Drobniewski F, Richter E, Rüsch-Gerdes S, et al. First evaluation after implementation of a quality control system for the second line drug susceptibility testing of Mycobacterium tuberculosis joint efforts in low and high incidence countries. PloS One. 2013;8: e76765. doi: 10.1371/journal.pone.0076765 24146924 PMC3795631

[8] World Health Organization. Anti-tuberculosis drug resistance in the world / the WHO/IUATLD Global Project on Anti-Tuberculosis Drug Resistance Surveillance, 1994–1997. Geneva: WHO; 1997. https://iris.who.int/handle/10665/64090

[9] World Health Organization. Anti-tuberculosis drug resistance in the world / the WHO/IUATLD Global Project on Anti-Tuberculosis Drug Resistance Surveillance. Report 2, Prevalence and trends. Geneva: WHO; 2000. https://iris.who.int/handle/10665/66493

[10] World Health Organization. Anti-tuberculosis drug resistance in the world: third global report / the WHO/IUATLD Global Project on Anti-Tuberculosis Drug Resistance Surveillance, 1999–2002. Geneva: WHO; 2004. https://iris.who.int/handle/10665/43103

[11] World Health Organization. Anti-tuberculosis drug resistance in the world: fourth global report. / the WHO/IUATLD Global Project on Anti-Tuberculosis Drug Resistance Surveillance, 2002–2007. Geneva: WHO; 2008. https://iris.who.int/handle/10665/43889

[12] World Health Organization. Optimized broth microdilution plate methodology for drug susceptibility testing of Mycobacterium tuberculosis complex. Geneva; 2022. https://www.who.int/publications-detail-redirect/9789240047419

[13] Schön T, Miotto P, Köser CU, Viveiros M, Böttger E, Cambau E. Mycobacterium tuberculosis drug-resistance testing: challenges, recent developments and perspectives. Clin Microbiol Infect. 2017;23: 154–160. doi: 10.1016/j.cmi.2016.10.022 27810467

[14] McDERMOTT W, Tompsett R. Activation of pyrazinamide and nicotinamide in acidic environments in vitro. Am Rev Tuberc. 1954;70: 748–754. doi: 10.1164/art.1954.70.4.748 13197751

[15] World Health Organization. Technical manual for drug susceptibility testing of medicines used in the treatment of tuberculosis. Geneva: WHO; 2018. https://www.who.int/publications-detail-redirect/9789241514842

[16] Zhang Y, Permar S, Sun Z. Conditions that may affect the results of susceptibility testing of Mycobacterium tuberculosis to pyrazinamide. J Med Microbiol. 2002;51: 42–49. doi: 10.1099/0022-1317-51-1-42 11800471

[17] Hoffner S, Angeby K, Sturegård E, Jönsson B, Johansson A, Sellin M, et al. Proficiency of drug susceptibility testing of Mycobacterium tuberculosis against pyrazinamide: the Swedish experience. Int J Tuberc Lung Dis. 2013;17: 1486–1490. doi: 10.5588/ijtld.13.0195 24125455

[18] Werngren J, Sturegård E, Juréen P, Ängeby K, Hoffner S, Schön T. Reevaluation of the critical concentration for drug susceptibility testing of Mycobacterium tuberculosis against pyrazinamide using wild-type MIC distributions and pncA gene sequencing. Antimicrob Agents Chemother. 2012;56: 1253–1257. doi: 10.1128/AAC.05894-11 22203587 PMC3294906

[19] Mok S, Roycroft E, Flanagan PR, Montgomery L, Borroni E, Rogers TR, et al. Overcoming the Challenges of Pyrazinamide Susceptibility Testing in Clinical Mycobacterium tuberculosis Isolates. Antimicrob Agents Chemother. 2021;65: e0261720. doi: 10.1128/AAC.02617-20 33972244 PMC8284449

[20] Horne DJ, Pinto LM, Arentz M, Lin S-YG, Desmond E, Flores LL, et al. Diagnostic accuracy and reproducibility of WHO-endorsed phenotypic drug susceptibility testing methods for first-line and second-line antituberculosis drugs. J Clin Microbiol. 2013;51: 393–401. doi: 10.1128/JCM.02724-12 23152548 PMC3553871

[21] Leonard B, Coronel J, Siedner M, Grandjean L, Caviedes L, Navarro P, et al. Inter- and intra-assay reproducibility of microplate Alamar blue assay results for isoniazid, rifampicin, ethambutol, streptomycin, ciprofloxacin, and capreomycin drug susceptibility testing of Mycobacterium tuberculosis. J Clin Microbiol. 2008;46: 3526–3529. doi: 10.1128/JCM.02083-07 18701659 PMC2566109

[22] Kim SJ. Drug-susceptibility testing in tuberculosis: methods and reliability of results. Eur Respir J. 2005;25: 564–569. doi: 10.1183/09031936.05.00111304 15738303

[23] World Health Organization. Policy Guidance on Drug-Susceptibility Testing (DST) of Second-Line Antituberculosis Drugs. Geneva: WHO; 2008. http://www.ncbi.nlm.nih.gov/books/NBK310860/26290924

[24] Salfinger M, Ahmedov S. Has the time come to discontinue proficiency testing? Int J Tuberc Lung Dis. 2009;13: 1193. 19793421

