# Supplementary figures and images for: Proficiency of phenotypic drug susceptibility testing for Mycobacterium tuberculosis in China, 2008–2021
Source: PLoS One. 2024 May 29;19(5):e0304265. doi: 10.1371/journal.pone.0304265 (PMC11135779; doi:10.1371/journal.pone.0304265)

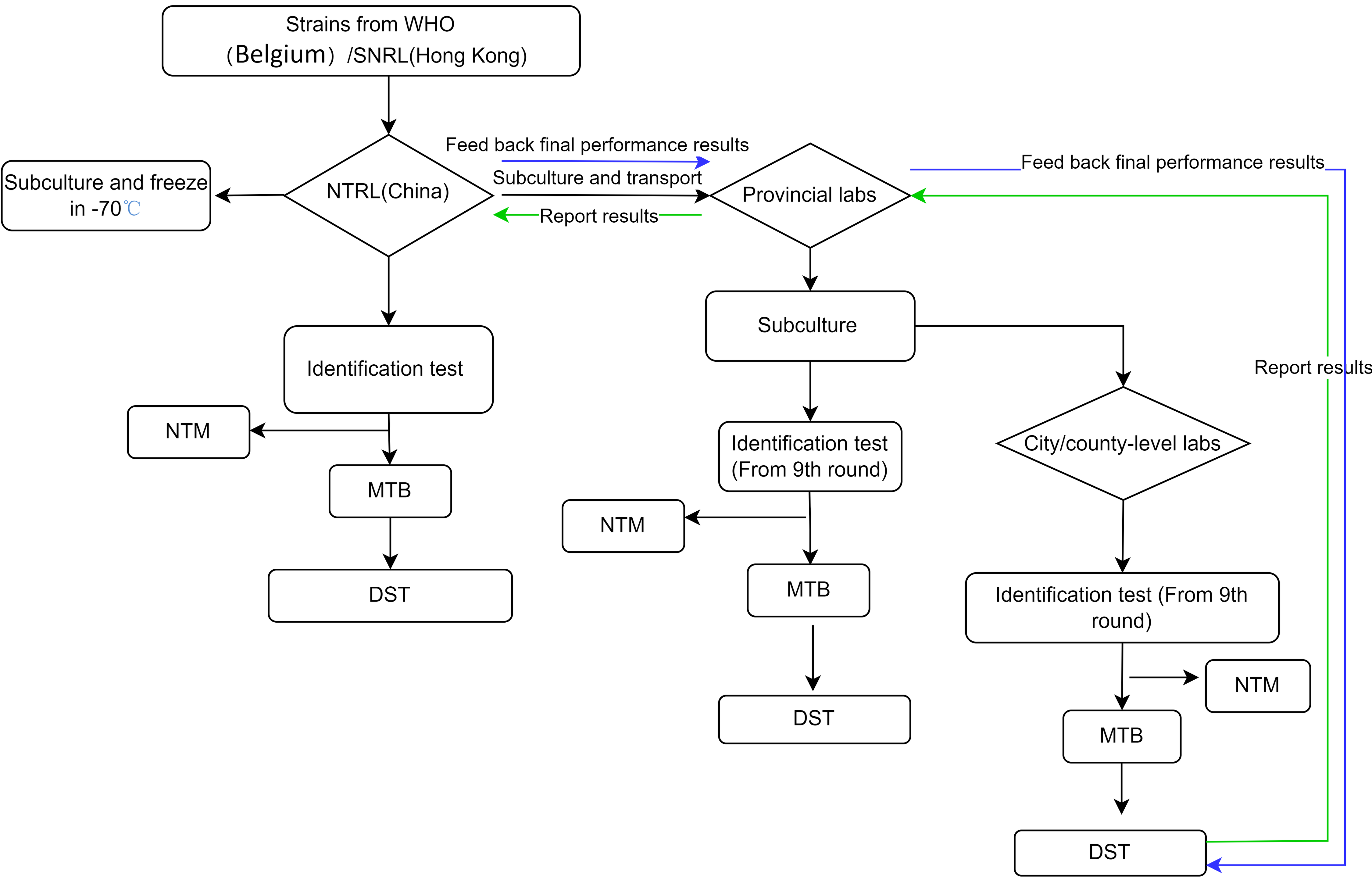

Supplement: S1 Fig — The flowchart contained laboratory testing, result reporting and feedback process. Identification test included biochemical test or rapid immunochromatographic assay (such as Capillia TB) is required to confirm MTB before DST since the 9th round. The standard results report form based on Excel is used to enter and report results from lower level labs to upper level labs as shown by green line. Excel software is used and then transferred to python since the 12th round for analysis of the performance indicators by NTRL and feedback the final performance results to provincial level labs. The provincial labs will feedback the results to lower level labs as shown by blue line. NTRL: National Tuberculosis Reference Laboratory; MTB: Mycobacterium Tuberculosis; NTM: Non-Tuberculous Mycobacteria; DST: Drug susceptibility testing. (TIF) [file pone.0304265.s001.tif]
